# Effectiveness of Self-Collected, Ambient Temperature–Preserved Nasal Swabs Compared to Samples Collected by Trained Staff for Genotyping of Respiratory Viruses by Shotgun RNA Sequencing: Comparative Study

**DOI:** 10.2196/32848

**Published:** 2023-11-24

**Authors:** Raymond Soto, Litty Paul, Christina A Porucznik, Heng Xie, Rita Czako Stinnett, Benjamin Briggs, Matthew Biggerstaff, Joseph Stanford, Robert Schlaberg

**Affiliations:** 1 Department of Family and Preventive Medicine University of Utah Salt Lake City, UT United States; 2 IDbyDNA Salt Lake City, UT United States; 3 Influenza Division Centers for Disease Control and Prevention Atlanta Georgia

**Keywords:** genotyping, self-collected nasal swabs, RNA sequencing, respiratory virus surveillance, surveillance, respiratory virus, influenza virus, pandemic, preparedness, testing capacity, self-test, viral genome analysis, swabs, barriers, early detection, nasal swab, temperature, public health, specimen, collection, diagnosis, laboratory, respiratory, virus, COVID-19

## Abstract

**Background:**

The SARS-CoV-2 pandemic has underscored the need for field specimen collection and transport to diagnostic and public health laboratories. Self-collected nasal swabs transported without dependency on a cold chain have the potential to remove critical barriers to testing, expand testing capacity, and reduce opportunities for exposure of health professionals in the context of a pandemic.

**Objective:**

We compared nasal swab collection by study participants from themselves and their children at home to collection by trained research staff.

**Methods:**

Each adult participant collected 1 nasal swab, sampling both nares with the single swab, after which they collected 1 nasal swab from 1 child. After all the participant samples were collected for the household, the research staff member collected a separate single duplicate sample from each individual. Immediately after the sample collection, the adult participants completed a questionnaire about the acceptability of the sampling procedures. Swabs were placed in temperature-stable preservative and respiratory viruses were detected by shotgun RNA sequencing, enabling viral genome analysis.

**Results:**

In total, 21 households participated in the study, each with 1 adult and 1 child, yielding 42 individuals with paired samples. Study participants reported that self-collection was acceptable. Agreement between identified respiratory viruses in both swabs by RNA sequencing demonstrated that adequate collection technique was achieved by brief instructions.

**Conclusions:**

Our results support the feasibility of a scalable and convenient means for the identification of respiratory viruses and implementation in pandemic preparedness for novel respiratory pathogens.

## Introduction

Challenges to global control of SARS-CoV-2 have underscored the need for accurate, safe, and rapidly scalable laboratory testing to inform rapid diagnosis and implementation of appropriate clinical and public health measures. Furthermore, early detection of emerging infectious disease threats is critical to limit spread. Over the last 4 decades, 1-3 newly emerging pathogens have been identified annually [1]. The risk of emerging infections is increasing, in part due to closer proximity of humans to wildlife and expanding geographic ranges of insect and arthropod vectors [2,3]. Outbreaks of diseases caused by respiratory tract infections, such as COVID-19, severe acute respiratory syndrome (SARS), Middle East respiratory syndrome (MERS), pandemic H1N1 influenza, and avian influenza, have specifically illustrated the global health burden posed by respiratory viruses with RNA genomes, prompting the implementation of public health policies and efforts to monitor and contain outbreaks [4,5]. Due to the genetic diversity of respiratory viral pathogens and the overlap between syndromes associated with distinct viruses, contemporary disease control efforts generally rely on a combination of methods, including rapid identification of emerging viral strains through targeted polymerase chain reaction (PCR)–based methods coupled with sequencing-based methods to enable molecular epidemiologic tracking of transmission. These activities can be streamlined by new methods of viral detection, such as metagenomic next-generation sequencing (NGS), to simultaneously detect and sequence respiratory viruses from human specimens [6].

Lower per-sample sequencing costs, accessible results through user-friendly analysis tools, and rapid turnaround of sequencing data have led to a growing number of diagnostic and public health laboratories applying NGS-based pathogen detection and profiling, which has enhanced and accelerated the detection and surveillance of emerging viruses [7-9]. Reliable detection of respiratory viruses requires robust methods for sample collection and preservation of viral RNA during transport because poor sample quality can lead to missed identification of significant pathogenic viruses [10]. Traditionally, laboratory diagnosis of respiratory viral infections has relied on collection of nasopharyngeal swabs by health care providers and uninterrupted cold chain transport to testing laboratories. In a previous study, evaluation of nasopharyngeal swabs from individuals with a known respiratory infection by metagenomic RNA sequencing showed high diagnostic agreement with a commercially available reverse transcription–PCR (RT-PCR) viral respiratory panel in a clinical laboratory setting, generated partial or full-length sequences in 86% of known positive samples, and detected additional viruses compared to the commercial panel [11].

However, community- and household-based sampling strategies offer potential advantages over sampling by health care workers in preparation for and during an outbreak. Specifically, anterior nasal swabs represent a noninvasive means for the detection and profiling of respiratory viruses via molecular diagnostic methods, provided appropriate measures to maintain sample integrity are taken [12-14]. Furthermore, nasal swabs can be collected at home by patients or their family members, limiting opportunities for further transmission from exposed individuals within health care facilities. Recent work has demonstrated high percent positive agreement in the detection of SARS-CoV-2 by targeted RT-PCR from self-collected nasal swabs compared to health care provider–collected nasopharyngeal swabs [15-17]. The feasibility of self-collected nasal swabs for detection of pathogen carriage and infection has also been previously demonstrated in an outpatient clinic setting for PCR-based detection of influenza from symptomatic patients [12,14]. Studies to date have focused on PCR-based detection of specific pathogens, and relatively few studies have directly compared the yield from participants to trained researchers or clinical workers.

In this feasibility study, we assessed detection of respiratory viruses by shotgun RNA sequencing using a room temperature–stable at-home nasal swab sampling kit, comparing swabs collected by participants to swabs collected by trained research staff. We also determined the acceptability to participants of collecting nasal swabs from their children and potential barriers to collection at home.

## Methods

### Study Participants

The inclusion criteria were families with children younger than 7 years who lived in Salt Lake County (to facilitate the required home visits) and in which the parent was able to read English. Invitation letters were sent to 34 families that were currently part of an ongoing cohort study (the Utah Children’s Project). In addition, a request for volunteers was posted on the Utah Children’s Project Facebook page. In total, 32 families responded, of which 4 declined to participate, 4 were not eligible, and 3 were eligible but not able to schedule a visit in the time window of the study. Thus, 21 families were enrolled over a period of 7 weeks from February to April 2018. This number of families was based on logistical considerations, without any power calculations. Study surveys were collected by paper questionnaire during the scheduled home visit to collect participants’ age, gender, ethnicity, race, household composition, and feedback on the sample collection process. The questionnaire was not pilot-tested or otherwise validated.

### Ethical Considerations

All aspects of this study were approved and carried out in accordance with the University of Utah Institutional Review Board (IRB_00086963**)**.

### Nasal Swab Collection

After the enrollment questionnaire was completed, participants were given a packet containing the necessary instructions and materials to collect the nasal swab samples from 1 adult and 1 child in each home. Swab collection kits containing DNA/RNA Shield HydraFlock swabs and 1-mL collection vials with self-centering caps (Puritan) were used for collection of nasal swab samples. The research staff member also provided a link to an existing video demonstrating collection procedures and access to an iPad (Apple) to view the video immediately prior to sample collection. Participants were given the opportunity and time to independently review the instructions, collect the nasal swab samples, and package them for mailing. Adult parents collected their own sample and separately collected the sample for their child. During this time, research staff were present but did not aid or guide in self-collection. Each participant collected 1 nasal swab and sampled both nares with the single swab. Once all the participant samples were collected for the household, the research staff member collected a separate single duplicate sample from each individual, again sampling both nares. Samples collected by research staff were transported at ambient temperature and transferred to a –80 °C freezer on the same day. Samples were labeled with IDs corresponding to the following information: family ID (F01-F21), participant type (A: adult; C: child), and collection type (S: self-collected; R: research staff–collected).

### Questionnaires

At the beginning of the home study visit, adult participants completed a pen and paper enrollment questionnaire about demographics and household characteristics. Questions were taken from the National Children’s Study enrollment questionnaire [18]. At the end of the home study visit, adult participants completed a brief pen and paper experience questionnaire about the collection of the sample, which included questions previously used in the National Children’s Study. Questions regarding nasal swab self-collection were generated specifically for this study and were not otherwise validated prior to this study.

### RNA Extraction, Next-Generation Sequencing, and Data Analysis

RNA was extracted using the ZymoBIOMICS DNA/RNA Miniprep kit (Zymo Research). cDNA libraries were prepared using the Trio RNA-Seq kit (NuGEN Technologies), quantified by quantitative-PCR using the KAPA library quantification kit (Roche), and sequenced on a NextSeq500 instrument (Illumina) to a depth of approximately 15 million 150-base-pair reads per sample. Sequencing data were demultiplexed allowing for no mismatches in the barcode sequences. Adapter sequences were trimmed, and the data were quality-filtered. Respiratory viruses were detected and viral genome sequences were assembled using the Explify Platform (v2, IDbyDNA). For phylogenetic analyses, complete human metapneumovirus (HMPV) genome sequences were obtained from GenBank. Multiple sequence alignments were performed with Geneious, and neighbor-joining trees were constructed with the Tamura-Nei method and bootstrapped using 500 replicates (Geneious v11.1.5).

### Statistical Analysis

Proportions and 95% CIs were calculated. Agreement between self-collected and staff-collected samples was assessed by Cohen κ statistic, which was estimated using Prism version 8.3.0 (GraphPad).

## Results

### Description of Study Participants

The demographics of the study participants are presented in Table 1. Of the adult participants, 85.7% (18/21) were female, while 52.4% (11/21) of children were female. The mean age was 32 (SD 3) years for adults and 3 (SD 2) years for children. The majority of participants self-identified as White (38/42, 90.5%) and of non-Hispanic/Latino ethnicity (41/42, 97.6%**)**. Most adult participants (17/21, 80.9%) indicated they had a bachelor’s degree or higher. Of the 21 families, 10 (47.6%) reported a recent upper respiratory illness, but the exact time frame was not defined.

**Table 1:** Demographics of enrolled participants.

		Adults (n=21)	Children (n=21)
**Sex, n**			
	Female	18	11
	Male	3	10
Age (years), mean (SD)		32 (3)	3 (2)
**Ethnicity, n**			
	Non-Hispanic/Latino	21	20
	Hispanic/Latino	0	1
**Race, n**			
	Asian	1	1
	White	19	19
	White and Asian	1	1
**Education, n**			
	Some college	4	N/A^a^
	Bachelor’s degree	7	N/A
	Graduate degree	10	N/A

^a^N/A: not applicable.

### Feedback on the Self-Collection Experience

Most adult participants (18/21, 85.5%) collected nasal swab samples with no (11/21, 52.4%) or little (7/21, 33.3%) self-reported difficulty (Table 2). All enrolled adult participants indicated that they would feel comfortable collecting nasal swab samples based on the provided instructions, and 95.2% (20/21) reported that felt they could mail the collected samples back without problems (Table 2). However, in this study, samples were transported by research staff. Most adult participants (18/21, 86%) preferred both video and written instructions.

**Table 2:** Adults’ responses to the collection experience survey.

Question		Responses (n=21), n
**Did you experience any difficulties in collecting the sample today?**		
	Not at all	11
	A little	7
	Some	3
	A lot	0
**Could you have collected this sample if there were no study staff present, using only the instruction materials provided?**		
	Yes	21
	No	0
**Could participant have followed instructions to mail samples back without problems:**		
	Yes	20
	Unsure	1
**If it were available, would participant have preferred:**		
	Video and written instructions	16
	Written instructions only	3
	Video instructions only	2
**In your opinion, in a real pandemic influenza study that involved nasal swabs, which household members would be willing to participate?**		
	Myself	19
	Other adults	20
	Children	17

### RNA-seq Data Quality and RNA Composition

**Figure 1:**
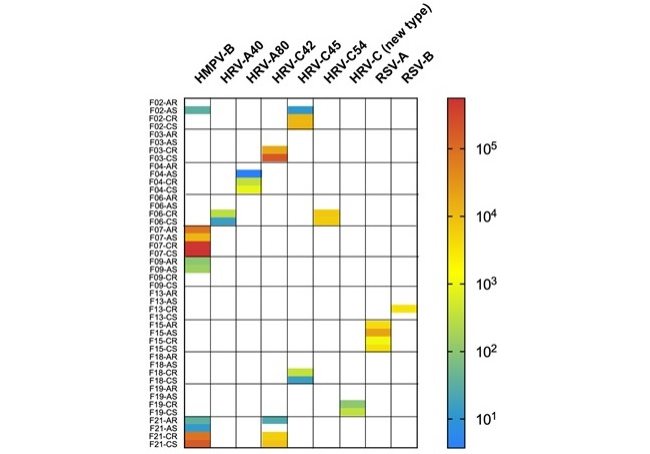
Detection of respiratory viruses by RNA sequencing. The color code indicates the number of normalized viral reads. Rows represent different samples and columns represent the viruses detected. Sample IDs convey the following information: family ID (F01-F21), participant type (A: adult; C: child), and collection type (S: self-collected; R: research staff–collected). HMPV: human metapneumovirus; HRV: human rhinovirus; RSV: respiratory syncytial virus.

A median of 9.05 (IQR 8.21-10.2) million sequencing reads were generated per sample. Respiratory viruses were detected in members of 52% (11/21) of families, including 6/10 (60%) families that had reported recent illness (Figure 1). Children tested positive in 10 families and adults tested positive in 6 families. In total, 9 different respiratory viruses were detected: human metapneumovirus type B (HMPV-B; detected in F02, F07, F09, and F21); respiratory syncytial virus type A (RSV-A; detected in F15); respiratory syncytial virus type B (RSV-B; detected in F13); human rhinovirus (HRV) types A40 (detected in F06), A80 (detected in F04), C42 (detected in F03 and F21), C45 (detected in F02 and F18), and C54 (detected in F06); and a diverse HRV-C most similar to HRV-C36 (detected in F19). In 2 children, 2 different respiratory viruses were detected (HRV-A40 and HRV-C54 in F06-C and HMPV-B and HRV-C56 in F21-C). Similarly, in 1 adult, 2 different respiratory viruses were detected (HMPV-B and HRV-C45 in F02-A). With 3 exceptions, the same viral status (including the status of absence of any virus) was identified in the staff- and self-collected swabs from the same participant (39/42, 93% agreement; κ=0.84, 95% CI 0.66-1.00). In 2 participants (F13-C and F21-A), RSV-B and HRV-C42 were detected only in the staff-collected swabs (Figure 1). In both cases, low concentrations of virus were detected and attempts to detect the same virus in the matching self-collected swabs by RT-PCR failed, consistent with the absence of viral detection in those self-collected samples. In a third participant (F02-A), 2 viruses (HMPV-B and HRV-C45) were detected only in the self-collected swab.

**Figure 2:**
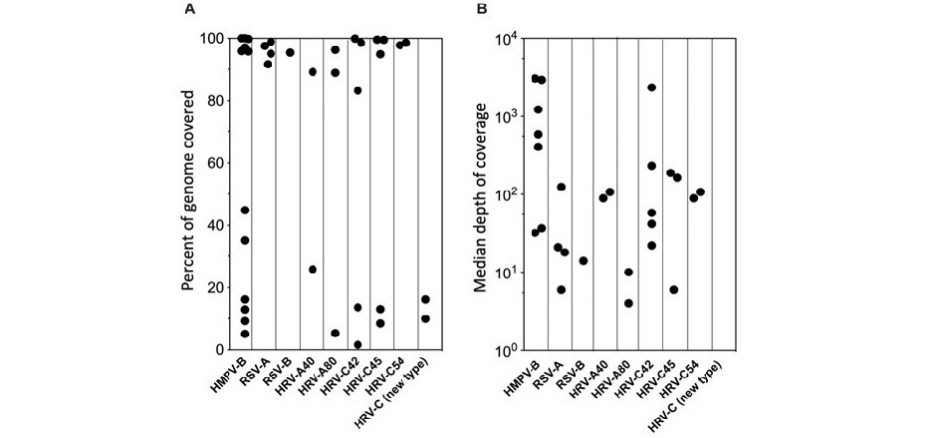
Summary of RNA sequencing depth of coverage. (A) Percentage of full-length assembly achieved for viral genomes. (B) Depth of coverage for each detected viral sample. HMPV: human metapneumovirus; HRV: human rhinovirus; RSV: respiratory syncytial virus.

Partial or full-length viral genome sequences with >80% coverage could be determined in 63% (22/35) of total detections. Among the samples for which depth of coverage could be calculated, the median depth of coverage was 89-fold (IQR 21-222; Figure 2). Concordance in viral subtype was evaluated in those cases where NGS detected the same viral pathogen with sufficient coverage of viral genomes from paired staff- and self-collected swabs. Results were consistent with the identification of the same strain in all cases with sufficient genome coverage. In sample pairs from 2 adult study participants (F09-A and F21-A), there was insufficient coverage to make a full comparison, despite agreement in virus detection between staff- and self-collected samples.

In one family (F14), the putative detection of an additional virus (human parainfluenza virus 4b) was suggested by sequencing reads that mapped to an identical reference genome in the self-collected adult swab (F14-AS) and in the staff-collected child swab (F14-CR); however, in both cases, genome coverage did not meet previously established criteria for positive diagnostic identification.

## Discussion

In this pilot, proof-of-concept study, we found that respiratory samples (anterior nasal swabs) self-collected from adults and children who were trained via online video and written instructions had comparable detection of respiratory viruses to samples concurrently collected by trained research personnel, with transport at ambient temperatures, as assessed by unbiased detection of respiratory viruses by NGS. All participants found the home collection procedures acceptable, and 85% reported minimal or no difficulty collecting the samples.

Our findings are consistent with prior studies that have found home collection of anterior nasal swabs to be acceptable in general populations, with high levels of good-quality samples [13,19-24]. Our results are also consistent with a growing body of studies indicating high agreement between self-collected swabs in adults and children (by parents) and swabs collected by health care workers or research personnel; κ ≥0.85 has been reported for the detection of several respiratory viruses, including influenza viruses, SARS-CoV-2, and metapneumovirus [12,13,17,25-30]. To our knowledge, this study is the first to evaluate agreement in self-collected versus researcher-collected nasal swabs specifically for shotgun RNA sequencing.

We considered any risk of nasal injury by self-collection to be minimal and outweighed by the potential benefit of understanding the feasibility and acceptability of self-collection of nasal samples for future research and public health surveillance. In one large, community-based study of over 4000 individuals given home collection kits with nasal swabs to be used on themselves or family members, there was one mild adverse event that did not require any medical attention and had no long-term impacts [31].

This method could be applied to test exposed or symptomatic individuals without the need for visits to a health care facility during outbreaks of emerging respiratory viruses [24,30,32]. In addition, it could streamline surveillance for known or emerging infectious disease threats by application to sentinels. Prior studies have supported the contributions of self-collected swab submission for molecular testing as a supplement to passive and syndromic surveillance for acute respiratory infections [11,14,19,20,32,33]. Guidance from the United States Food and Drug Administration (FDA) to permit the submission of self-collected swabs for SARS-CoV-2 testing illustrates the advantages of this strategy to reduce opportunities for transmission from infected individuals to health care workers and vulnerable populations and to mitigate bottlenecks associated with sample collection by providers [34]. The nasal swab collection kits used in this study have a shelf life of 3 years and stabilize samples at ambient temperature for up to 30 days. This allows for economical stocking and transport of collection kits during an outbreak or in support of surveillance efforts.

This study had several limitations. The sample size was small in this pilot study. Recent respiratory illness was self-reported and no information was collected about the duration of symptoms or the time between onset of symptoms and sample collection. It is therefore possible that individuals may no longer have been shedding virus at the time of sampling. Diagnostic performance of metagenomic sequencing was not compared to a reference method (eg, FDA-cleared PCR panels for the detection of respiratory pathogens), although high agreement has been previously reported [11].

We detected a diverse range of respiratory viruses, determined viral genome sequences in the majority of cases, and showed that viral genomes were identical in staff- and self-collected swabs. Our identification of a divergent strain of HRV-C with only 74% nucleotide sequence identity with the most similar known strain demonstrates the power of unbiased pathogen detection by metagenomics in the context of the well-described diversity of rhinoviruses [35,36]. This suggests an advantage of sequencing-based surveillance for generating viral genomes for high-resolution molecular typing. Studies in hospitalized patients have shown that this genotypic information can assist in efforts to contain transmission [37-39].

In summary, our findings support a growing body of evidence that at-home nasal swab kits for viral detection can be reliably used, are well-tolerated, and provide a scalable solution for the detection and profiling of respiratory viruses. In addition to prior work showing the reliability of self-collected nasal swabs with PCR-based diagnostic tests, our findings suggest that self-collected nasal swabs can be reliable for shotgun RNA sequencing. As metagenomics is integrated into routine diagnostic workflows in a rapidly growing number of laboratories, pathogen genotypic information generated as part of diagnostic testing can be more easily shared and is likely to prove valuable in support of surveillance research and public health applications.

## Abbreviations

FDA: Food and Drug Administration
HMPV: human metapneumovirus
HRV: human rhinovirus
MERS: Middle East respiratory syndrome
NGS: next-generation sequencing
PCR: polymerase chain reaction
RSV: respiratory syncytial virus
RT-PCR: reverse transcription–polymerase chain reaction
SARS: severe acute respiratory syndrome

## References

[1] Woolhouse M, Gaunt E (2007). Ecological origins of novel human pathogens. Crit Rev Microbiol.

[2] (2014). The U.S. government and global emerging infectious disease preparedness and response. The Henry J Kaiser Family Foundation.

[3] Plowright RK, Parrish CR, McCallum H, Hudson PJ, Ko AI, Graham AL, Lloyd-Smith JO (2017). Pathways to zoonotic spillover. Nat Rev Microbiol.

[4] Saunders-Hastings PR, Krewski D (2016). Reviewing the history of pandemic influenza: understanding patterns of emergence and transmission. Pathogens.

[5] Tang JW, Lam TT, Zaraket H, Lipkin WI, Drews SJ, Hatchette TF, Heraud J, Koopmans MP, INSPIRE investigators (2017). Global epidemiology of non-influenza RNA respiratory viruses: data gaps and a growing need for surveillance. Lancet Infect Dis.

[6] Schlaberg R, Chiu CY, Miller S, Procop GW, Weinstock G, Professional Practice Committee and Committee on Laboratory Practices of the American Society for Microbiology, Microbiology Resource Committee of the College of American Pathologists (2017). Validation of metagenomic next-generation sequencing tests for universal pathogen detection. Arch Pathol Lab Med.

[7] Besser J, Carleton HA, Gerner-Smidt P, Lindsey RL, Trees E (2018). Next-generation sequencing technologies and their application to the study and control of bacterial infections. Clin Microbiol Infect.

[8] Motro Y, Moran-Gilad J (2017). Next-generation sequencing applications in clinical bacteriology. Biomol Detect Quantif.

[9] Deurenberg RH, Bathoorn E, Chlebowicz MA, Couto N, Ferdous M, García-Cobos S, Kooistra-Smid AMD, Raangs EC, Rosema S, Veloo ACM, Zhou K, Friedrich AW, Rossen JWA (2017). Application of next generation sequencing in clinical microbiology and infection prevention. J Biotechnol.

[10] Goldberg B, Sichtig H, Geyer C, Ledeboer N, Weinstock GM (2015). Making the leap from research laboratory to clinic: challenges and opportunities for next-generation sequencing in infectious disease diagnostics. mBio.

[11] Graf EH, Simmon KE, Tardif KD, Hymas W, Flygare S, Eilbeck K, Yandell M, Schlaberg R (2016). Unbiased detection of respiratory viruses by use of RNA sequencing-based metagenomics: a systematic comparison to a commercial PCR panel. J Clin Microbiol.

[12] Akmatov MK, Gatzemeier A, Schughart K, Pessler F (2012). Equivalence of self- and staff-collected nasal swabs for the detection of viral respiratory pathogens. PLoS One.

[13] Dhiman N, Miller RM, Finley JL, Sztajnkrycer MD, Nestler DM, Boggust AJ, Jenkins SM, Smith TF, Wilson JW, Cockerill FR, Pritt BS (2012). Effectiveness of patient-collected swabs for influenza testing. Mayo Clin Proc.

[14] Elliot AJ, Powers C, Thornton A, Obi C, Hill C, Simms I, Waight P, Maguire H, Foord D, Povey E, Wreghitt T, Goddard N, Ellis J, Bermingham A, Sebastianpillai P, Lackenby A, Zambon M, Brown D, Smith GE, Gill ON (2009). Monitoring the emergence of community transmission of influenza A/H1N1 2009 in England: a cross sectional opportunistic survey of self sampled telephone callers to NHS Direct. BMJ.

[15] Hanson K, Barker A, Hillyard D, Gilmore N, Barrett J, Orlandi R, Shakir SM (2020). Self-collected anterior nasal and saliva specimens versus health care worker-collected nasopharyngeal swabs for the molecular setection of SARS-CoV-2. J Clin Microbiol.

[16] Tu Y, Jennings R, Hart B, Cangelosi GA, Wood RC, Wehber K, Verma P, Vojta D, Berke EM (2020). Swabs collected by patients or health care workers for SARS-CoV-2 testing. N Engl J Med.

[17] McCulloch DJ, Kim AE, Wilcox NC, Logue JK, Greninger AL, Englund JA, Chu HY (2020). Comparison of unsupervised home self-collected midnasal swabs with clinician-collected nasopharyngeal swabs for detection of SARS-CoV-2 infection. JAMA Netw Open.

[18] Landrigan PJ, Trasande L, Thorpe LE, Gwynn C, Lioy PJ, D'Alton ME, Lipkind HS, Swanson J, Wadhwa PD, Clark EB, Rauh VA, Perera FP, Susser E (2006). The national children's study: a 21-year prospective study of 100,000 American children. Pediatrics.

[19] Haussig JM, Targosz A, Engelhart S, Herzhoff M, Prahm K, Buda S, Nitsche A, Haas W, Buchholz U (2019). Feasibility study for the use of self-collected nasal swabs to identify pathogens among participants of a population-based surveillance system for acute respiratory infections (GrippeWeb-Plus)-Germany, 2016. Influenza Other Respir Viruses.

[20] Jackson ML, Nguyen M, Kirlin B, Madziwa L (2015). Self-collected nasal swabs for respiratory virus surveillance. Open Forum Infect Dis.

[21] Ricci S, Lodi L, Citera F, Nieddu F, Moriondo M, Guarnieri V, Giovannini M, Indolfi G, Resti M, Zanobini A, Azzari C (2021). How home anterior self-collected nasal swab simplifies SARS-CoV-2 testing: new surveillance horizons in public health and beyond. Virol J.

[22] Zoch-Lesniak B, Ware RS, Grimwood K, Lambert SB (2020). J Pediatric Infect Dis Soc.

[23] Muñoz-Ramírez S, Escribano-López B, Rodrigo-Casares V, Vergara-Hernández C, Gil-Mary D, Sorribes-Monrabal I, Garcés-Sánchez M, Muñoz-Del-Barrio MJ, Albors-Fernández AM, Úbeda-Sansano MI, Planelles-Cantarino M, Largo-Blanco E, Suárez-Vicent E, García-Rubio J, Bruijning-Verhagen P, Orrico-Sánchez A, Díez-Domingo J (2021). Feasibility of a hybrid clinical trial for respiratory virus detection in toddlers during the influenza season. BMC Med Res Methodol.

[24] Heimonen J, McCulloch DJ, O'Hanlon J, Kim AE, Emanuels A, Wilcox N, Brandstetter E, Stewart M, McCune D, Fry S, Parsons S, Hughes JP, Jackson ML, Uyeki TM, Boeckh M, Starita LM, Bedford T, Englund JA, Chu HY (2021). A remote household-based approach to influenza self-testing and antiviral treatment. Influenza Other Respir Viruses.

[25] Murray MA, Schulz LA, Furst JW, Homme JH, Jenkins SM, Uhl JR, Patel R, Cockerill FC, Myers JF, Pritt BS (2015). Equal performance of self-collected and health care worker-collected pharyngeal swabs for group a streptococcus testing by PCR. J Clin Microbiol.

[26] Emerson J, Cochrane E, McNamara S, Kuypers J, Gibson RL, Campbell AP (2013). Home self-collection of nasal swabs for diagnosis of acute respiratory virus infections in children with cystic fibrosis. J Pediatric Infect Dis Soc.

[27] Seaman CP, Tran LTT, Cowling BJ, Sullivan SG (2019). Self-collected compared with professional-collected swabbing in the diagnosis of influenza in symptomatic individuals: A meta-analysis and assessment of validity. J Clin Virol.

[28] Malosh RE, Petrie JG, Callear AP, Monto AS, Martin ET (2021). Home collection of nasal swabs for detection of influenza in the Household Influenza Vaccine Evaluation Study. Influenza Other Respir Viruses.

[29] Suntarattiwong P, Mott JA, Mohanty S, Sinthuwattanawibool C, Srisantiroj N, Patamasingh Na Ayudhaya O, Klungthong C, Fernandez S, Kim L, Hunt D, Hombroek D, Brummer T, Chotpitayasunondh T, Dawood FS, Kittikraisak W, PRIME Study Group (2021). Feasibility and performance of self-collected nasal swabs for detection of influenza virus, respiratory syncytial virus, and human metapneumovirus. J Infect Dis.

[30] Woodall CA, Thornton HV, Anderson EC, Ingle SM, Muir P, Vipond B, Longhurst D, Leeming JP, Beck CR, Hay AD (2021). Prospective study of the performance of parent-collected nasal and saliva swab samples, compared with nurse-collected swab samples, for the molecular detection of respiratory microorganisms. Microbiol Spectr.

[31] Kim A, Brandstetter E, Wilcox N, Heimonen J, Graham C, Han P, Starita LM, McCulloch DJ, Casto AM, Nickerson DA, Van de Loo MM, Mooney J, Ilcisin M, Fay KA, Lee J, Sibley TR, Lyon V, Geyer RE, Thompson M, Lutz BR, Rieder MJ, Bedford T, Boeckh M, Englund JA, Chu HY (2021). Evaluating specimen quality and results from a community-wide, home-based respiratory surveillance study. J Clin Microbiol.

[32] Uyeki TM, Bernstein HH, Bradley JS, Englund JA, File TM, Fry AM, Gravenstein S, Hayden FG, Harper SA, Hirshon JM, Ison MG, Johnston BL, Knight SL, McGeer A, Riley LE, Wolfe CR, Alexander PE, Pavia AT (2019). Clinical practice guidelines by the infectious diseases society of America: 2018 update on diagnosis, treatment, chemoprophylaxis, and institutional outbreak management of seasonal influenzaa. Clin Infect Dis.

[33] Brendish NJ, Malachira AK, Armstrong L, Houghton R, Aitken S, Nyimbili E, Ewings S, Lillie PJ, Clark TW (2017). Routine molecular point-of-care testing for respiratory viruses in adults presenting to hospital with acute respiratory illness (ResPOC): a pragmatic, open-label, randomised controlled trial. Lancet Respir Med.

[34] (2020). Coronavirus (COVID-19) Update: FDA, Gates Foundation, UnitedHealth Group, Quantigen, and U.S. Cotton Collaborate to Address Testing Supply Needs. U.S. Food & Drug Aministration.

[35] Lamson D, Renwick N, Kapoor V, Liu Z, Palacios G, Ju J, Dean A, St George K, Briese T, Lipkin WI (2006). MassTag polymerase-chain-reaction detection of respiratory pathogens, including a new rhinovirus genotype, that caused influenza-like illness in New York State during 2004-2005. J Infect Dis.

[36] Lau SKP, Yip CCY, Tsoi H, Lee RA, So L, Lau Y, Chan K, Woo PCY, Yuen K (2007). Clinical features and complete genome characterization of a distinct human rhinovirus (HRV) genetic cluster, probably representing a previously undetected HRV species, HRV-C, associated with acute respiratory illness in children. J Clin Microbiol.

[37] Payne DC, Biggs HM, Al-Abdallat MM, Alqasrawi S, Lu X, Abedi GR, Haddadin A, Iblan I, Alsanouri T, Al Nsour M, Sheikh Ali S, Rha B, Trivedi SU, Rasheed MAU, Tamin A, Lamers MM, Haagmans BL, Erdman DD, Thornburg NJ, Gerber SI (2018). Multihospital outbreak of a Middle East respiratory syndrome coronavirus deletion variant, Jordan: a molecular, serologic, and epidemiologic investigation. Open Forum Infect Dis.

[38] Houlihan CF, Frampton D, Ferns RB, Raffle J, Grant P, Reidy M, Hail L, Thomson K, Mattes F, Kozlakidis Z, Pillay D, Hayward A, Nastouli E (2018). Use of whole-genome sequencing in the investigation of a nosocomial influenza virus outbreak. J Infect Dis.

[39] Li C, Li W, Zhou J, Zhang B, Feng Y, Xu C, Lu Y, Holmes EC, Shi M (2020). High resolution metagenomic characterization of complex infectomes in paediatric acute respiratory infection. Sci Rep.

